# The role of structural order in heterogeneous ice nucleation†

**DOI:** 10.1039/d1sc06338c

**Published:** 2022-04-08

**Authors:** Gabriele C. Sosso, Prerna Sudera, Anna T. Backes, Thomas F. Whale, Janine Fröhlich-Nowoisky, Mischa Bonn, Angelos Michaelides, Ellen H. G. Backus

**Affiliations:** Department of Chemistry, University of Warwick Gibbet Hill Road Coventry CV4 7AL UK g.sosso@warwick.ac.uk; Max Planck Institute for Polymer Research Ackermannweg 10 55128 Mainz Germany; Max Planck Institute for Chemistry Hahn-Meitner-Weg 1 55128 Mainz Germany; Yusuf Hamied Department of Chemistry, University of Cambridge Lensfield Road Cambridge CB2 1EW UK; Department of Physical Chemistry, University of Vienna Währingerstrasse 42 1090 Wien Austria

## Abstract

The freezing of water into ice is a key process that is still not fully understood. It generally requires an impurity of some description to initiate the heterogeneous nucleation of the ice crystals. The molecular structure, as well as the extent of structural order within the impurity in question, both play an essential role in determining its effectiveness. However, disentangling these two contributions is a challenge for both experiments and simulations. In this work, we have systematically investigated the ice-nucleating ability of the very same compound, cholesterol, from the crystalline (and thus ordered) form to disordered self-assembled monolayers. Leveraging a combination of experiments and simulations, we identify a “sweet spot” in terms of the surface coverage of the monolayers, whereby cholesterol maximises its ability to nucleate ice (which remains inferior to that of crystalline cholesterol) by enhancing the structural order of the interfacial water molecules. These findings have practical implications for the rational design of synthetic ice-nucleating agents.

## Abstract 2

The freezing of water into ice is still not fully understood. Here, we investigate the role of structural disorder within the biologically relevant impurities that facilitate this fundamental phase transition.

## Graphical abstract

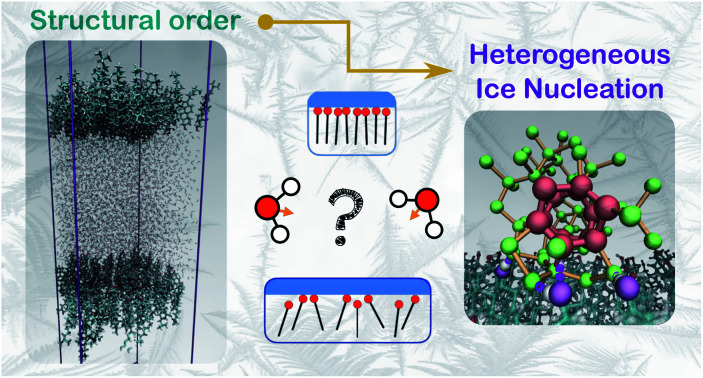

## Introduction

The formation of ice from supercooled liquid water is one of the most important phase transitions: it plays a role in global phenomena such as climate change^1–3^ and it is key to transformative medical treatments such as regenerative and reproductive medicine,^4–7^ as the storing of biological material relies on cryopreservation protocols where it is essential to control the extent of ice formation in cells.

In nature, ice almost always forms thanks to the help of impurities which lower the free energy cost of creating an ice nucleus large enough to grow into an actual ice crystal.^1,8^ This process is known as heterogeneous nucleation, and it exploits specific impurities, which range from mineral dust^1,9–11^ to bacterial fragments.^12–16^ However, what exactly makes such substances effective in facilitating the nucleation of ice remains to be fully understood.

In recent years, both experiments and simulations have made substantial progress in the field.^8,10,17–29^ For instance, recent evidence suggests that the activity of a given ice-nucleating agent (INA) might be due to an interplay between the order within said agent (*e.g.*, its crystalline, ordered nature, or the presence of specific defects) and its molecular structure, particularly the presence of specific functional groups (the hydroxyl –OH group is a common example).^30–34^ However, which characteristic – the degree of order or the presence of functional groups – is most important, is unknown, as disentangling the two different contributions is a challenge for both experiments and simulations.^8,34^ In fact, more often than not, we seek to gain insight into ice nucleation by comparing the effectiveness of different materials, thus acting on both order and molecular structure at the same time.

Here, we answer this crucial question by adopting a different approach, namely probing the ice-nucleating ability of the same substance, cholesterol (CHL), in different forms, from its crystalline phase^35^ to self-assembled monolayers,^36,37^ characterised by a variable degree of order and flexibility. Cholesterol is an interesting model system to study since it is an effective ice-nucleating agent and a number of steroids with molecular structure similar to CHL show equally strong ice-nucleating ability in their crystalline phases.^38,39^ In this work, we have isolated the impact of structural order only, by systematically investigating the ice-nucleating ability of the very same compound, cholesterol, from the crystalline (and thus ordered) form to disordered self-assembled monolayers, thus focusing in detail on the impact of structural order on heterogeneous ice nucleation without having to compare the ice-nucleating efficiency of different materials.

By bringing together state-of-the-art experimental techniques and atomistic computer simulations, we find that CHL monolayers display a weaker ice-nucleating activity compared to CHL crystals. A systematic investigation of CHL monolayers characterised by different coverages (and thus different degrees of structural order as well as flexibility) reveals that the degree of order of the CHL molecules within the monolayers has a direct impact on the population of pre-critical ice nuclei forming at the CHL–water interfaces. In particular, there exists a specific interval of CHL coverages that maximises the ability of this steroid to order interfacial water molecules in such a way as to facilitate ice nucleation.

This systematic investigation of the impact of order and disorder on the ice-nucleating ability of a biologically relevant compound allows us to unravel the microscopic motivations underpinning its activity and offer valuable practical guidelines toward the rational design of the next generation of INAs. In particular, our results imply that more emphasis should be placed on the supra-molecular aspects of biological INAs as opposed to their molecular structure.

## Methods

### Droplet freezing assays

Freezing experiments of the CHL monolayer were performed using the high-throughput twin-plate ice nucleation assay (TINA).^40^ Droplets of pure water (30 μL volume) were pipetted into sterile 384-well plates (Eppendorf, Hamburg, Germany) by a liquid handling station (epMotion ep5073, Eppendorf). Pure water was prepared as described in ref. 40. To obtain monolayers of CHL on top of the pure water droplets, 1 μL aliquots of CHL in chloroform at different concentrations were pipetted on top of the pure water droplets using a liquid handling station. The concentration of the chloroform solutions ranged from 0.046 mM to 4.69 mM, corresponding to a surface area ranging from 400 Å^2^ mol^−1^ to 4 Å^2^ mol^−1^, respectively, assuming that all cholesterol remains at the water surface and does not diffuse into the bulk. Of each concentration, 96 droplets were measured, and experiments were performed from 0 °C to −30 °C at a cooling rate of 1 °C min^−1^. The temperature uncertainty was ±0.2 °C.^40^ As controls, 30 μL droplets of pure water, and 30 μL droplets of pure water with 1 μL droplets of pure chloroform were measured. From the fraction of total droplets frozen, we have computed the cumulative number of nucleation sites per cholesterol molecule, referred to as *n*_mol_, which allows us to compare the ice-nucleating ability of the different CHL monolayers with that of CHL crystals. The details of the *n*_mol_ analysis can be found in the ESI.† The higher the value of *n*_mol_, the stronger the ice nucleation activity of the sample (per molecule). Confidence intervals were calculated using Monte Carlo simulations according to ref. 41, based on the assumption that the number of freezing events in a temperature interval is expected to follow a Poisson distribution. For each experiment, the data was divided into temperature intervals of 0.5 K width. The observed number of events in each temperature interval was taken as the expectation value for the number of events, and 1000 Poisson distributed random numbers were generated for each temperature interval. This effectively gives 1000 independent possible experimental outcomes, which were used to calculate possible *n*_mol_ values. The confidence intervals are the 10th to 90th percentile range of the simulated values. Due to the relatively high number of droplets used in these experiments the confidence interval is relatively narrow.

### Molecular dynamics simulations

Cholesterol and water molecules were both modelled at the atomistic level *via* the CHARMM36 force field^42,43^ and the TIP4P/Ice^44^ model, respectively. This specific combination has been validated on multiple occasions within the recent literature: not only has it been demonstrated to provide an accurate description of supercooled water and ice at the interface with biological material,^45–47^ but we have also explicitly verified its reliability for water and ice in contact with cholesterol.^33^

The GROMACS package (version 5.1.4)^42,48^ has been used to perform molecular dynamics (MD) simulations within a variety of ensembles, including the N*γ*T ensemble (with constant surface tension *γ*)^49^ so as to take into account different CHL coverages (*i.e.*, different values of surface area per molecule, *S*_A_/mol). A leap-frog algorithm^50^ has been used to integrate Newton's equations of motion with a time step of 2 fs. A twin cutoff of 12 Å has been used for both electrostatic and van der Waals interactions, where, for the latter, forces have been smoothly switched to zero between 10 and 12 Å. The Bussi–Donadio–Parrinello thermostat^51^ has been used to sample the canonical ensemble, in conjunction with the Berendsen barostat (in a semi-isotropic fashion given the slab geometry)^52^ if performing simulations in the NPT or N*γ*T ensemble. The coupling constants for the thermostat and barostat are 0.5/1.0 (CHL/water) and 4.0 ps, respectively. The LINCS^53^ and SETTLE^54^ algorithms have been used to constrain the CHL bonds involving hydrogen atoms and to enforce the geometry of water molecules, respectively.

To simulate self-assembled CHL monolayers at the interface with water, we have used the setup depicted in Fig. 2a: the symmetry of the system along its *z*-axis (parallel to the long edge of the simulation box which has been extended according to the guidelines of ref. 55) is such that it eliminates spurious electrostatic effects due to the intrinsic dipole moment of CHL molecules. 2D periodic boundaries conditions have been employed to deal with this slab-like computational setup, in conjunction with the Bostick and Berkowitz Ewald summation scheme.^55^

**Fig. 1:**
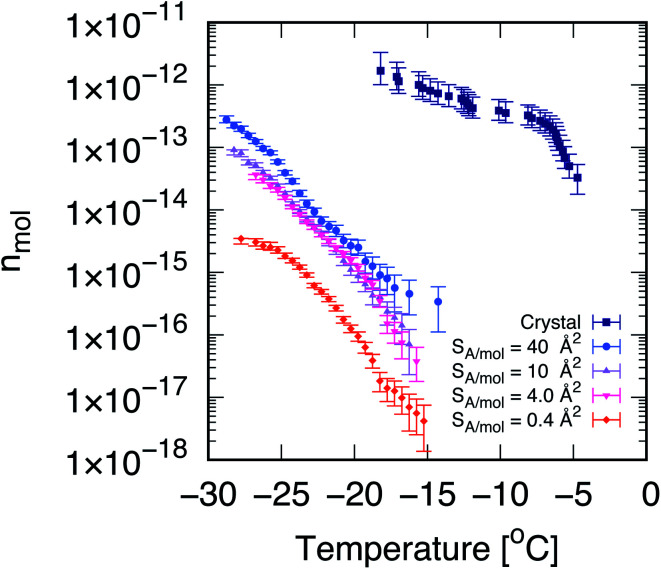
Cholesterol crystals are much more active ice-nucleating agents than cholesterol monolayers. Cumulative number of nucleation sites (*n*_mol_) as a function of temperature, measured *via* droplet fraction frozen experiments for CHL monohydrate crystals (crystal, from ref. 33) as well as for self-assembled CHL monolayers characterised by different values of surface area per molecule, *S*_A_/mol. The volume of the water droplets in contact with CHL crystals and monolayers is 1 μL and 30 μL, respectively. The uncertainty, in terms of temperature, associated with the data for CHL crystals and monolayers is ±0.4 and 0.2 K, respectively.

**Fig. 2:**
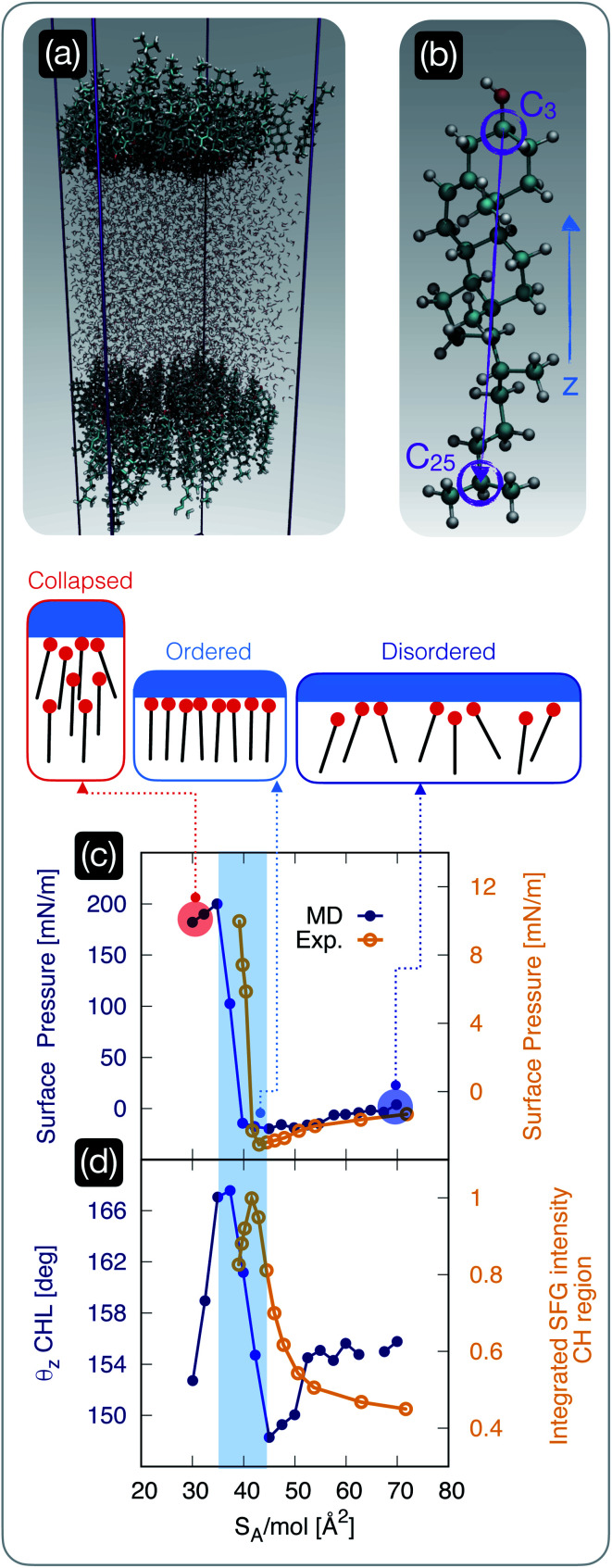
The structure of cholesterol monolayers dramatically changes as a function of surface pressure. (a) A representative snapshot of a molecular dynamics simulation used to model self-assembled cholesterol monolayers in contact with water: the hydrophilic heads of the cholesterol molecules within two monolayers point toward the water phase. (b) Atomistic model of a cholesterol molecule: the vector connecting the C_25_ to the C_3_ carbon atoms forms an angle *θ*_*z*_ with the *z*-axis of the simulation box; carbon, hydrogen, and oxygen atoms are depicted in light blue, white, and red, respectively. (c) Surface pressure (from molecular dynamics simulations, “MD” [blue]) and experimentally measured surface tension (“Exp” [yellow]) as a function of surface area per molecule *S*_A_/mol; the region of the plot shaded in light blue highlights the sharp rise in the surface pressure/tension below a critical value of *S*_A_/mol, beyond which the monolayer collapses. (d) The *θ*_*z*_ angle defined in panel (b) (from molecular dynamics simulations [blue]) and the experimentally measured square root of the (integrated) intensity of the sum-frequency generation spectroscopy signal corresponding to the C–H stretch region [yellow] (see the ESI† for further details); the same shaded region defined in panel (c) highlights the abrupt ordering of the cholesterol molecules within the monolayer in a specific interval of *S*_A_/mol.

The (*xy*) in-plane dimensions of the simulation box along the compression protocol ranged from 67 (*S*_A_/mol ∼ 70 Å^2^) to 44 Å (*S*_A_/mol ∼ 30 Å^2^). The *z*-dimension of the simulation box was set to 200 Å, roughly three times the extent of the CHL/water system along the *z* direction, thus allowing for a substantial vacuum region as suggested by the protocol detailed in ref. 55. The water layer consisted of 4752 water molecules, which resulted in a minimum thickness (at *S*_A_/mol ∼ 70 Å^2^) of roughly 40 Å. We note that even at the lowest value of *S*_A_/mol we have simulated, the in-plane dimensions of the simulation box are significantly larger than (twice) the extent of the pre-critical ice nuclei we have observed,^56^ which can exceptionally span up to 16 Å in any given direction.

We have also considered a setup involving a single layer of CHL molecules, a situation that might be problematic because of spurious effects related to nonphysical electric fields within the simulation box.^57–60^ However, as discussed in detail in the ESI,† the results are perfectly consistent with the setup featuring two layers of CHL molecules in contact with the upper and lower part of the water slab – the thickness of which is enough to guarantee bulk-like structural properties of water within the middle of it.

Different coverages of CHL self-assembled monolayers were achieved as follows: 64 CHL molecules were randomly positioned at a distance of 3 Å from a water slab equilibrated within the NPT ensemble at ambient temperature and pressure. The minimum distance between the CHL molecules in the *xy*-plane was 5 Å. A series of N*γ*T simulations enforcing different values of *γ* were then performed to gradually increase the *S*_A_/mol – we are thus computing surface pressure *vs.* area isotherms *via* a compression protocol. Each of these simulations lasted a minimum of 50 ns, which we have verified is sufficiently long to converge the structural properties of the system at room temperature. We note that when performing N*γ*T simulations with GROMACS, the *z*-component of the pressure and the surface tension are coupled *via* a pressure bath that can only be implemented *via* a Berendsen barostat.

A quantity of special interest when dealing with self-assembled monolayers at aqueous interfaces is the surface pressure, which in this case can be obtained as:

1 *π* = *γ*_water–vacuum_ − 2*γ*_water–CHL–vacuum_,

where *γ*_water–vacuum_ is the surface tension of pure water and *γ*_water–CHL–vacuum_ is the surface tension of the CHL-coated water–vacuum interface. The factor two is needed to take into account the fact that we have two water–CHL–vacuum interfaces within our simulation setup. *γ*_water–vacuum_ has been calculated for the TIP4P/Ice water model at ambient pressure and temperature in ref. 61 (*γ*_water–vacuum_ = 80.1 mN m^−1^), while *γ*_water–CHL–vacuum_ can be written as:2
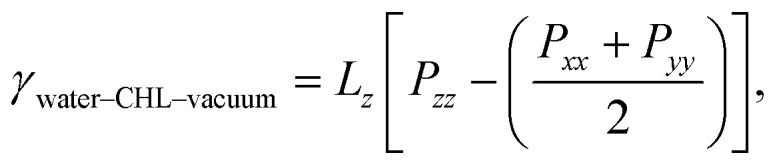
where *L*_*z*_ and *P*_*αα*_ correspond to the length of the simulation box along the *z*-axis and the diagonal component of the pressure tensor along the *α* coordinate.

NVT simulations were performed at selected values of *S*_A_/mol in order to eliminate the effect of pressure fluctuations: after 50 ns of equilibration, each configuration has been quenched from 300 to 230 K in 50 ns following a linear quenching protocol. These configurations have finally been used as the starting point for the long (≈1.4 μs) production runs used to obtain the results reported in this work to study the formation of ice nuclei. We note that we validated this quenching protocol in ref. 33, where we also pinpointed 230 K as the ideal supercooling (*S* = *T*_M_ − *T* = 40 K) to investigate icy water for the TIP4P/Ice water model (the melting temperature *T*_m_ of TIP4P/Ice is 270 ± 3 K).^62^

Molecules belonging to ice nuclei have been identified thanks to a clustering algorithm based on the local order parameter introduced by Wang *et al.*,^63^ which in turn leverages the so-called Steinhardt order parameters.^64^ We have recently reviewed the full procedure, based on the open-source PLUMED software^65^ in ref. 66: a specific example including the relevant input files can be found in a dedicated entry of the so-called PLUMED “NEST”.^67^

### Sum frequency generation spectroscopy

#### SFG setup

The SFG experiments were performed using a Ti–sapphire regenerative amplifier setup (Spitfire Pro, Spectra-Physics) generating 800 nm pulses with a repetition rate of 1 kHz and a duration of ∼40 fs. Part of the output was used to generate broadband infrared pulses in an optical parametric amplifier (TOPAS-C, Light Conversion) with subsequent difference frequency generation. The IR pulse energy at the sample was approximately 3 μJ. The other part of the laser output was spectrally narrowed to 15 cm^−1^ using a Fabry–Perot etalon. The resulting 800 nm beam had a pulse energy of 20 μJ. All sample spectra were collected in SSP (SFG-visible-IR) polarization with incident angles of 34° and 36° for the visible and IR beam, respectively. To reduce heating effects from the laser, the trough containing the CHL monolayer on water was continuously rotated (see ref. 68). To prevent oxidation during the experiment, the sample area was purged with nitrogen. The sample SFG spectra were divided by an SFG spectrum from *z*-quartz to account for the IR laser pulse spectral content. All spectra were acquired for 10 minutes.

#### Sample preparation

For the SFG experiments, CHL (Sigma Aldrich) was dissolved in chloroform to a concentration of 0.64 mM; all solutions were prepared in a glove box to avoid sample oxidation. To form a monolayer on water, a well-defined number of drops were deposited onto the water surface to get a specific surface coverage (calculated using the trough surface area and the number of molecules in a drop, assuming cholesterol does not diffuse into bulk water), using a microliter syringe (from Hamilton) with a droplet size of 0.5 μL. The surface pressure was monitored throughout the experiment using a Kibron surface tensiometer. The CHL layer was monitored *via* Brewster angle microscopy as well (further details can be found in the ESI†).

## Results

### Comparing the ice-nucleating ability of crystals and monolayers

Droplet freezing assays provide a quantitative indication of the ice-nucleating ability of different substances. We have employed a site-specific, or singular interpretation of our ice nucleation data to facilitate background subtraction and comparison between measurements on crystalline cholesterol and the cholesterol monolayers. Details of this analysis are provided in the ESI.† In Fig. 1, we report the cumulative number of ice nucleation sites (*n*_mol_, see the Methods section) for CHL crystals^33^ and for self-assembled CHL monolayers characterised by different values of *S*_A_/mol. It is evident that the same compound shows a much stronger ice-nucleating ability in its crystalline phase, while in the form of a self-assembled monolayer, its potency is greatly reduced.

In addition, the ice-nucleating activity of CHL crystals shows a two-step trend (see Fig. 1) which is far less pronounced in the case of CHL monolayers. We note that the cumulative number of ice-nucleating sites reported for both biological ice-nucleating agents such as Snowmax^69^ and inorganic ice-nucleating agents such as the mineral feldspar^70^ often span a similar range of temperature compared to crystalline CHL, with a sharp increase at the onset of nucleation followed by a plateauing at stronger supercooling. This two-step trend is likely caused by different nucleation sites on the surface of CHL crystals, specifically on the (001) hydroxylated surface and potentially active at different degrees of supercooling (as discussed in detail in ref. 33). In the case of CHL monolayers, however, within a certain range of *S*_A_/mol (as discussed in the next section) the CHL–water interface can be considered as largely homogeneous – *i.e.*, there are no defects to be found, as opposed to most crystalline surfaces. Identifying the exact morphology of ice-nucleating sites on crystalline substrates is beyond the capabilities of existing instrumentation (see *e.g.* ref. 34). However, based on our previous results^33^ and the topology of the crystalline surface,^71^ we argue that the different nucleation sites on cholesterol crystals are probably due to steps or similar surface defects. As such, these results suggest that, for certain surface coverages at least, self-assembled monolayers offer a single, well-defined substrate for ice to nucleate upon – in stark contrast with the diverse variety of nucleation sites typically observed in the case of crystalline INAs.^10,72,73^

It is also clear that the ice-nucleating activity of CHL monolayers depends on their coverage, which in turn is inversely proportional to their *S*_A_/mol. As illustrated in Fig. 1, at very high coverages (*e.g.*, 0.4 Å^2^ mol^−1^) the ice-nucleating ability of CHL monolayers is basically negligible: this regime corresponds to a situation where CHL monolayers “collapse”^74^ (as confirmed by the MD simulations reported in the next section, see Fig. 2). In contrast, at the lower coverage of *S*_A_/mol = 4 Å^2^ mol^−1^, the activity of CHL monolayers is increased, and indeed we observe similar ice-nucleating activity for *S*_A_/mol = 10 and 40 Å^2^ mol^−1^. However, for values of *S*_A_/mol greater than 10 Å^2^ mol^−1^, the ice-nucleating activity of CHL monolayers could not be distinguished from that of the background, despite the fact that their ice nucleating activity in terms of *n*_mol_ is stronger than what we observe for *S*_A_/mol = 4 Å^2^ mol^−1^. This apparent contradiction is clarified in the ESI (Fig. S7†). It is important to note that entirely eliminating the effect of the background (in this case, chloroform, see the Methods section) is extremely challenging with droplet freezing assays,^75,76^ particularly when dealing with compounds displaying relatively weak ice-nucleating activity such as that of CHL monolayers.

Thus, in order to probe whether there exists a “sweet spot” in terms of the ice-nucleating activity of CHL monolayers, within the range of *S*_A_/mol that are basically inaccessible by means of droplet freezing experiments (*i.e. S*_A_/mol greater or equal than 10 Å^2^ mol^−1^), we have brought together MD simulations and SFG experiments. As discussed in the next sections, such a sweet spot does exist, and it originates from the changes in the microscopic structure of the monolayers at different coverages.

### The structural order of cholesterol monolayers depends strongly on coverage

Having established that the ice-nucleating ability of CHL monolayers is not only inferior to that of CHL crystals, but also depends on *S*_A_/mol, we focus on the degree of order within the CHL monolayers as a function of surface pressure. To this end, we employ both MD simulations and sum-frequency generation (SFG) spectroscopy.

We start by investigating the surface pressure of the CHL monolayers–water system as a function of *S*_A_/mol. The computational setup is depicted in Fig. 2a and involves two CHL monolayers in contact with a slab of water (see the Methods section). The MD result is compared in Fig. 2c with the surface pressure we have measured experimentally, according to the protocol described in the ESI.† While the relatively small size of our simulation boxes, especially in the low-*S*_A_/mol regime, leads to a substantial overestimation of the absolute values of the surface pressure, the qualitative features of the isotherm are in excellent agreement with the experimental data (which in turn are consistent with previous results, see, *e.g.*, ref. 74). In particular, both experiments and simulations pinpoint ∼40 Å^2^ mol^−1^ as a critical value of *S*_A_/mol below which the surface pressure increases sharply: this particular value of *S*_A_/mol (also known as the “lift off area”) has been reported in several previous experimental works^36,37,77,78^ and is due to the monolayer becoming increasingly compact until it collapses. In particular, Rapaport *et al.*^74^ have observed, at 36 Å^2^ mol^−1^, the formation of a film composed of a trilayer, with a rough upper surface, a smooth, highly crystalline bilayer in the middle, and a more disordered monolayer in contact with the water phase. However, the finite number of CHL molecules in MD simulations only form a disordered monolayer (see Fig. S8 in the ESI†), which could explain the mismatch in the absolute values of the simulated surface pressure when compared with the experimental results (albeit previous measurements reported values of surface pressure in the 0–50 mN m^−1^ range even upon the collapse).

Importantly, the range of *S*_A_/mol reported in Fig. 2c and d corresponds to coverages that yield very similar ice-nucleating activity, if measured *via* the droplet freezing assay discussed in the previous section. We also note that for high values of *S*_A_/mol (*i.e.*, lower surface coverage), both experiments and simulations identify negative values of surface pressure. This could be interpreted, in principle, as an indication of metastability (*i.e.*, CHL molecules are to some extent repelled by the interface) that has been previously reported in the case of low coverages of lipids, polymers and proteins^79–81^ – but never in the case of CHL.^36,37,77,78^ However, as in some conditions, we have observed the same effect experimentally even in the absence of CHL (as discussed in the ESI†), we conclude that the negative pressure might also be interpreted as an effect of finite size effects and surface tension mismatch in the simulations – and evaporation in the experimental case.

In order to identify potential structural changes within the CHL monolayers, it is useful to define a molecular axis for CHL molecules. As illustrated in Fig. 2b, this is defined as the vector connecting the C_25_ and C_3_ carbon atoms. This choice is not unique, but we have verified that different definitions of molecular axis do not significantly impact our results. Then, we define *θ*_*z*_ as the angle between the C_25_–C_3_ CHL molecular axis and the *z*-axis of the simulation box (*i.e.*, perpendicular to the CHL/water interfaces illustrated in Fig. 2a). As illustrated in Fig. 2d, the *S*_A_/mol region corresponding to rather compact monolayers (*S*_A_/mol = 35–45 Å^2^, see the shaded area in Fig. 2c and d) has a much higher degree of structural order compared to high values of *S*_A_/mol. This is demonstrated by the high average value of the *θ*_*z*_ angle, which indicates that in this “optimal” interval of *S*_A_/mol, the molecular axes of CHL molecules are on average perpendicular to the CHL–water interface. Interestingly, we note that the surface of interest for CHL crystals in the context of ice nucleation (which we have identified in ref. 33) is characterised by *S*_A_/mol = 38.44 Å^2^ mol^−1^, thus suggesting that indeed the *S*_A_/mol = 35–45 Å^2^ range might correspond to the most efficient packing density for CHL molecules. In fact, in this interval of *S*_A_/mol, it has been reported^74,82^ that CHL molecules are arranged in a monolayer characterised by trigonal symmetry. This monolayer is either very compact (as we approach the lift-off area) or characterised by relatively ordered domains that fail to cover the entire CHL–water interface. Our simulations are consistent with these features, as illustrated in Fig. S8 of the ESI,† where we discuss the morphology of the simulated CHL monolayers as a function of *S*_A_/mol in greater detail.

We note that, despite the fact that the surface density of the CHL monolayers at the “sweet spot” and that of CHL crystals is very similar, CHL monolayers are still not quite as ordered as a crystalline surface. As reported in Fig. S8 in the ESI,† the extent of trigonal order within the monolayers, even at the “sweet spot”, is limited to short/medium range. In addition, the monolayers are not as flat as a pristine CHL surface, and CHL molecules in monolayers can and will diffuse in-plane – as opposed to the crystal where their in-plane position is constrained by the crystalline structure. We note that the lack of long-range order, as well as the in-plane mobility of CHL molecules within the monolayers, give rise to an entirely different morphology from that of the crystal, for which surface defects, such as kinks, are likely responsible for the variety of nucleation sites, as can be inferred from the results reported in Fig. 1 for CHL crystals. Thus, the ice nucleating activity of CHL monolayers is, even at the “sweet spot”, far inferior to that observed for CHL crystals.

Our MD results are further supported by SFG spectroscopy measurements. The SFG spectra in the CH/OH stretch vibrational region, corresponding to different CHL coverages, can be found in the ESI† and show the most intense bands around 2850–2870 cm^−1^ and around 2950 cm^−1^. In line with ref. 83, these bands are most likely dominated by symmetric and asymmetric stretch vibration of the –CH_3_ groups in the tail at the C_25_ side of the molecule (see Fig. 2b). As in SSP polarization the projection of the dipoles on the *z*-axis are measured, both the asymmetric and symmetric –CH_3_ stretch mode will increase upon collective alignment.^84^ In Fig. 2d, the SFG signal integrated within the spectral region corresponding to the –CH orientation has been plotted. The increase of the integrated intensity, in line with the MD results, also suggests collective alignment of the CHL molecules within the monolayer. Further details about the structure of the CHL monolayers and the structure of the CHL–water interfaces (including density profiles and electric field distributions) can be found in the ESI,† together with SFG spectra corresponding to different CHL coverages. At this stage, the key question is whether the structure of the CHL monolayers, particularly the degree of order, has a direct impact on the nucleation of ice – a possibility we will investigate in the next section.

### Microscopic insight into the formation of ice nuclei

**Fig. 3:**
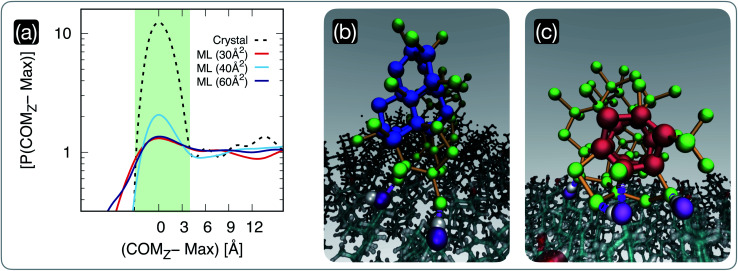
The population of pre-critical ice nuclei changes according to the structure of the cholesterol surface. (a) Probability density *P*(COM_*z*_) of the *z*-coordinate of the centre of mass of the largest nucleus found in each CHL–water configuration generated by means of long (0.5 μs) MD simulations. The positions of the peaks of these distributions, corresponding to the increase in the nuclei population at the CHL–water interface, have been aligned to zero. The distributions have also been normalised so that the baseline corresponding to the population of nuclei within the bulk of the water slab is equal to one. We report results for water on a CHL crystal (crystal, see ref. 33 for further details) and on three CHL monolayers characterised by *S*_A_/mol = 30, 40 and 60 Å^2^. The light green shaded region qualitatively highlights the width of the CHL–water interface. (b) and (c) Representative snapshots of pre-critical ice nuclei at the CHL–water interface (taken from an MD simulation of the *S*_A_/mol = 40 Å^2^ system): double-diamond and hexagonal cages (see text) are highlighted in blue and red, respectively. Oxygen atoms belonging to ice nuclei and their interatomic bonds are depicted in green and orange, respectively. The oxygen belonging to the CHL–OH groups hydrogen-bonded with the ice nuclei are highlighted in purple.

We have seen in the previous section that the degree of order within CHL monolayers changes dramatically according to their surface area per molecule. Crucially, our MD simulations reveal that these structural changes in the CHL monolayers translate into different propensities for (pre-critical) ice nuclei to form at the CHL–water interface, as illustrated in Fig. 3. In particular, we report in Fig. 3a the probability density *P*(COM_*z*_) of the *z*-coordinate of the centre of mass of the largest ice nucleus found in each configuration generated employing long (0.5 μs) MD simulations. The positions of the peaks corresponding to the increase in the nuclei population at the CHL–water interface have been aligned to zero. The distributions have also been normalised so that the baseline corresponding to the population of nuclei within the bulk of the water slab is equal to one. It is evident that, in the case of CHL crystals, there is a very strong tendency for the ice nuclei to form at the CHL–water interface (light blue shaded region in Fig. 3a) compared to the rest of the water slab. In fact, the *P*(COM_*z*_ − Max) in the interfacial region is about ten times higher than everywhere else. The situation is different for CHL monolayers, where this tendency for the nuclei to favour the CHL–water interfacial region strongly depends on the *S*_A_/mol. For very low values of *S*_A_/mol (30 Å^2^ in Fig. 3a) corresponding to very disordered monolayers beyond the collapse threshold, there is no apparent preference for the ice nuclei to form at the CHL–water interface. However, in the highly-ordered region (*S*_A_/mol = 40 Å^2^ in Fig. 3a), there is a significant propensity for the ice nuclei to form at the CHL–water interface, albeit to a much lesser extent than on the CHL crystal. Interestingly, as we move toward larger values of *S*_A_/mol (60 Å^2^ in Fig. 3a) corresponding to rather sparse and disordered monolayers, the difference in terms of *P*(COM_*z*_ − Max) between the interfacial region and the bulk of the water slabs decreases once more.

These results indicate that in this case, the analysis of the population of pre-critical nuclei^85^ is predictive of the ice-nucleating ability of the different systems under consideration. In fact, according to our experimental data (see Fig. 1), CHL crystals are more active as ice-nucleating agents than CHL monolayers, and this difference is clearly consistent with the data reported in Fig. 3a. While frozen droplet assays cannot assess the relative potency of the different CHL monolayers, due to their limited potency, our MD simulations allow us to pinpoint a specific range of *S*_A_/mol where CHL monolayers are most active. This “sweet spot” is at *S*_A_/mol = ∼40 Å^2^, which lies in between the low-coverage (high *S*_A_/mol) and collapsed (low *S*_A_/mol) regions, thus highlighting the correlation between the degree of order within the monolayers and their ice-nucleating activity.

Interestingly, when we examine the nature of the pre-critical nuclei at the level of supercooling considered in our MD simulations, there is a coexistence of cubic and hexagonal ice nuclei (a potential indication of the formation of stacking disordered ice^86–88^). As depicted in Fig. 3b and c, double-diamond cages (DDCs) and hexagonal cages (HCs), the building blocks of cubic and hexagonal ice respectively (see, *e.g.*, ref. 89 for further details) can both form at the CHL–water interface, aided by the formation of hydrogen bonds between water and the hydrophilic heads of CHL molecules. In particular, the –OH groups of CHL (purple spheres in Fig. 3b and c) are amphiphilic, as they can act as both hydrogen bond acceptors and donors. As such, the ice nuclei form in direct contact with the CHL, as opposed to what has been reported for, *e.g.*, some ice-nucleating proteins where a layer of “structured” water acts as a template for the ice nuclei to grow upon.^16,90^ In fact, the presence of the –OH amphiphilic functional groups seems to facilitate ice nucleation in a similar fashion to what we have previously reported for CHL crystals,^33^ where we have also observed the emergence of both cubic and hexagonal ice. In the case of CHL crystals, the symmetry of the –OH network within the amphiphilic heads of the cholesterol molecules is such that the ice nuclei can form a rather ordered ice–CHL interface, where on average one in two water molecules forms a hydrogen bond with the –OH groups of cholesterol.^33^ However, for CHL monolayers, even at the sweet spot the limited extent of symmetry as well as the inherent surface roughness of the system lead to the rather disordered ice–CHL interface depicted in Fig. 3b and c, where – whilst water molecules still form hydrogen bond with the –OH groups of cholesterol – it can be seen that fully-fledged DDCs and HCs form above the interfacial CHL–water layer.

### The structuring of water as seen by experiments and simulations

**Fig. 4:**
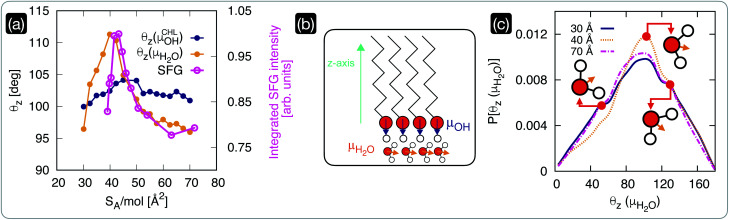
The structuring of interfacial water underpins the ice-nucleating abilities of cholesterol monolayers*.* (a) Comparison of the average orientation of the dipole moments (see panel (b)) of CHL (*θ*_*z*,CHL_) and water molecules as obtained *via* MD simulations with the square root of the integrated intensity of the experimental SFG signal in the –OH spectral region. (b) Schematics of the dipole moments of CHL and water defined with respect to the *z*-axis of the MD simulation box. (c) Probability density of the dipole moment for interfacial water molecules (*P*[*θ*_*z*_(*μ*_H_2_O_)], see text for the definition) at low and high coverage (*S*_A_/mol = 70 and 30 Å^2^, respectively), compared with the result obtained for a value of *S*_A_/mol (40 Å^2^) corresponding to the highly ordered region of CHL highlighted in Fig. 2; representative orientations of the water molecules are included as insets.

We have seen in the previous section that within the interval of surface area per molecule where cholesterol molecules are most ordered, the CHL monolayers have a strong ice-nucleating ability of CHL monolayers – albeit still inferior to that of CHL crystals (see Fig. 1). The microscopic mechanism underpinning this activity lies in the structuring of water molecules at the CHL–water interface, as summarised in Fig. 4. From our MD simulations, we can compute for different values of *S*_A_/mol the average value of the angle formed by the –OH fragments of either water or cholesterol molecules and the *z*-axis of our simulation box (Fig. 4b). Defining the extent of the CHL–water interface, and thus choosing which water molecules can be labelled as “interfacial water”, entails a certain degree of uncertainty (see, *e.g.*, ref. 91). Here, we have verified that defining the interfacial region boundary anywhere between 5 and 10 Å (the second peak of the density profile of water on CHL crystals sits ∼7 Å away from the substrate) provides consistent results. In order to compare our analysis with our previous work on CHL crystals,^33^ the results reported here have thus been obtained by identifying any water molecule within 7 Å of any oxygen atom belonging to CHL molecules as interfacial water.

The *θ*_*z*_ values obtained *via* our MD simulations are compared with the experimentally measured integrated SFG signal in the O–H stretch region (possibly originating from CHL and water) in Fig. 4a. The experimental SFG spectra, including phase-resolved SFG spectra, can be found in the ESI.† As the SFG spectra are measured in SSP polarization, the projection of the water dipole on the *z*-axis is measured. The integrated SFG intensity can thus directly be compared to the *θ*_*z*_ values of the MD simulation. Interestingly, our MD simulations allow us to disentangle the contributions of –OH fragments belonging to either water or CHL molecules. While *θ*_*z*_(*μ*^CHL^_OH_) displays only a marginal variation as a function of *S*_A_/mol, *θ*_*z*_(*μ*_H_2_O_) shows a sharp increase in the CHL-ordered region. This trend is in excellent agreement with the experimental data and demonstrates that the increase in the SFG signal is indeed due to the ordering of interfacial water as the CHL monolayers become more ordered.

Our simulations also offer the opportunity to investigate the changes in the orientation of interfacial water as a function of CHL coverage. In particular, we have chosen to compare the results obtained at low and high coverage (*S*_A_/mol = 70 and 30 Å^2^, respectively), with the result obtained for a value of *S*_A_/mol (40 Å^2^) corresponding to the highly ordered region of CHL illustrated in Fig. 2. As depicted in Fig. 4c, interfacial water molecules tend on average to sit on top of the CHL monolayers in a configuration (*θ*_*z*_(*μ*_H_2_O_) ∼ 102°) where one of the hydrogen atoms points toward the CHL surface, leaving the oxygen atom available to create a hydrogen bond with an –OH group of the CHL. A similar configuration produces a shoulder in the probability density at *θ*_*z*_(*μ*_H_2_O_) ∼ 130°, which remains unchanged with the value of *S*_A_/mol. However, the population of the feature at *θ*_*z*_(*μ*_H_2_O_) ∼ 55° substantially decreases for CHL coverages corresponding to the highly ordered region highlighted in Fig. 2, while the probability of finding water molecules in the *θ*_*z*_(*μ*_H_2_O_) ∼ 102° configuration increases. In the proximity of the sweet spot in terms of CHL *S*_A_/mol, interfacial water molecules are on average more likely to be able to fully leverage the amphiphilic character of the –OH group of CHL molecules. This is because the ∼102° orientation allows for either or both (a) a hydrogen bond between water and CHL where the water act as a donor, and; (b) a hydrogen bond between water and CHL where the water act as an acceptor. On the contrary, the ∼55° orientation only allows for a hydrogen bond between water and CHL where the water act as a donor. Thus, at the sweet spot, water molecules are on average more likely to interact more effectively with the CHL monolayer, which in turn facilitates the emergence of the OH (from CHL)–water rings network responsible for the formation of both double-diamond and hexagonal ice cages – in a similar fashion to what we have observed for crystalline CHL.^33^ This subtle orientational change leads to a higher degree of order, as illustrated in Fig. 4a.

Thus, it appears that in a specific interval of *S*_A_/mol (*i.e.*, a specific CHL coverage) CHL monolayers order interfacial water to an extent sufficient to trigger ice formation. This sweet spot, in terms of ice-nucleating activity, cannot be detected *via* the frozen droplet measurements reported in Fig. 1, as the activity of CHL monolayers is still relatively weak (and thus affected by the ice nucleating activity of the background) within the relevant range of *S*_A_/mol. However, both our MD and SFG results successfully identify this subtle trend, which is due to the fact that CHL molecules are clearly best packed and most ordered within the crystalline phase. The *S*_A_/mol of CHL monolayers can influence their ice-nucleating ability to a great extent, but their degree of order (which translates in the structuring of interfacial water) is still lower than that achieved by crystalline packing. We note that simulating a perfectly ordered monolayer, where the CHL molecules are constrained to their equilibrium positions within the crystalline structure of CHL, corresponds to the computational setup we have used in ref. 33 to investigate the ice-nucleating activity of CHL crystals. Thus, obtaining microscopic insight into the structure of interfacial water is key to understand the origins of heterogenous ice nucleation on self-assembled monolayers. In fact, the formation of more-or-less ordered monolayers of water at the interface between the ice-nucleating agent and the water phase has been recently debated in the context of whether or not the nucleation of ice follows a classical pathway in the case of *e.g.* phloroglucinol dihydrate monolayers^92^ as well as wurtzite-structured surfaces.^93^

## Conclusions

The freezing of water into ice is, at its very core, a question of which particular impurity can facilitate heterogeneous ice nucleation and how. Recent evidence suggests that amongst the different microscopic factors playing a role in this process, the interplay between the molecular structure and the degree of order of an impurity might be the key.^30–34^ However, it is not clear whether molecular structure outweighs order – or the other way around, chiefly because disentangling the two is a very challenging task for both experiments and simulations.

In this work, we have been able to tackle this open question by monitoring and rationalising the ice-nucleating ability of the same compound, cholesterol (CHL), from crystals to self-assembled monolayers with varying degrees of order.

We have found that CHL crystals outperform any self-assembled CHL monolayer we have investigated in terms of ice-nucleating ability. In addition, our results demonstrate that the more ordered these monolayers are, the better they can facilitate the formation of ice. In fact, we argue that an analysis of the pre-critical ice nuclei, as obtained by means of unbiased molecular dynamics simulations, can be used, in conjunction with experimental measurements such as SFG, to infer the actual ice-nucleating ability of a given material.

Crucially, conventional droplet freezing assays often struggle to identify useful trends when dealing with compounds with relatively weak ice-nucleating activity, such as CHL monolayers. One could argue that weakly active INA are of limited practical interest. However, identifying and understanding the changes in their ice-nucleating ability as a function of their structural properties is essential to uncover novel guidelines for the rational design of synthetic INAs.

In particular, by bringing together state of the art experimental and computational techniques, we have traced the origin of this ice-nucleating sweet spot to the molecular-level details of the CHL–water interface: less dense CHL monolayers are more disordered (especially if compared to the crystal phase) and less effective in modifying the ordering of interfacial water, which in turn enhances the ice-nucleating ability of the CHL substrate.

While this work focuses entirely on CHL, we expect our findings to be directly applicable to similar systems: for instance, particles containing long-chain fatty acids are of great relevance to the formation of ice driven by sea spray aerosols,^94^ and there are many examples of ice-nucleating impurities in the form of self-assembled monolayers of alcohols.^95–97^ In addition, the formation of ice in biological matter, which is key in the context of cryopreservation, does involve the formation of ice on/through the lipid bilayers which form the building blocks of cells.^98–101^ Control of ice nucleation very often improves the outcomes of cryopreservation procedures^76,102^ and, critically, the origin of ice formation inside cells remains very poorly understood.^103^ Improved understanding of the mechanism of heterogeneous ice nucleation by biological substances has the potential to both facilitate synthesis of new and effective biocompatible ice nucleators and shed light on the start of damaging intracellular ice formation.

Importantly, the observation that order plays an important role in heterogeneous ice nucleation, has a strong impact on the design and discovery of the next generation of ice-nucleating agents, many of which are bound to be biological in nature.^1,15,104–106^ In fact, some of them harness already supra-molecular structures with variable degrees of ordering (ice-nucleating proteins being an obvious example^107–111^) to facilitate ice formation. At the moment, there is a strong emphasis on the molecular structure of these compounds as opposed to their topology: our results suggest that we might want to shift our attention toward the design of supramolecular assemblies as opposed to trying to discover the most effective functional groups that are supposed to trigger ice formation – often a trial-and-error process.

Indeed, while playing with molecular structure is relatively straightforward (for instance, there is a substantial body of work devoted to the selective mutation of ice-nucleating proteins), acting on supra-molecular topology and particularly the degree of order within it is a much more complex task, with similar compounds displaying entirely different solid-phase morphologies, not to mention the incredibly challenging characterisation of the ice-nucleating sites in the case of crystalline ice-nucleating agents. While the solution of the puzzle offered by heterogeneous ice nucleation might not be within our reach yet, this investigation provides an essential piece that we hope will foster future work aimed at, *e.g.*, systematic studies of the ice-nucleating ability of supramolecular assemblies with a strong potential to be leveraged as effective ice-nucleating agents.

## Data availability

Data for this paper are available at: https://wrap.warwick.ac.uk/164520/.

## Author contributions

G. C. S. performed the molecular dynamics simulations. P. S. and E. H. G. B. performed the sum frequency generation spectroscopy measurements. A. T. B. performed the droplet freezing assays. All the authors contributed to the analysis and the interpretation of the results as well as to the writing of the manuscript.

## Conflicts of interest

There are no conflicts of interest to declare.

## Supplementary Material

SC-013-D1SC06338C-s001
