# Supplementary material for: The role of structural order in heterogeneous ice nucleation
Source: Chem Sci. 2022 Apr 8;13(17):5014–26. doi: 10.1039/d1sc06338c (PMC9067566; doi:10.1039/d1sc06338c)
Supplement: SC-013-D1SC06338C-s001 [file SC-013-D1SC06338C-s001.pdf]

Electronic Supplementary Material (ESI) for Chemical Science.
This journal is © The Royal Society of Chemistry 2022

The Role of Structural Order in Heterogeneous Ice Nucleation

SUPPLEMENTARY INFORMATION

Gabriele C. Sosso,[*,†] Prerna Sudera,[‡] Anna T. Backes,[¶] Thomas F. Whale,[†] Janine Fröhlich-Nowoisky,[¶] Mischa Bonn,[‡] Angelos Michaelides,[§] and Ellen H.G. Backus[‡,∥]

†*Department of Chemistry, University of Warwick, Gibbet Hill Road, Coventry CV4 7AL, United Kingdom*

‡*Max Planck Institute for Polymer Research, Ackermannweg 10, 55128 Mainz, Germany*

¶*Max Planck Institute for Chemistry, Hahn-Meitner-Weg 1, 55128 Mainz, Germany*

§*Yusuf Hamied Department of Chemistry, University of Cambridge, Lensfield Road, Cambridge CB2 1EW, United Kingdom*

∥*Department of Physical Chemistry, University of Vienna, Währingerstrasse 42, 1090 Wien, Austria*

E-mail: g.sosso@warwick.ac.uk

We provide supplementary material with further details on:

- The structure of the cholesterol(CHL)-water interface as a function of S_A/mol.
- The computational geometry, particularly the comparison between the results obtained by simulating mono- or bi-layers of CHL molecules.
- The experimental measurements of the surface pressure as a function of S_A/mol.
- Brewster Angle Microscopy Measurements
- SFG spectra as a function of CHL coverage
- Phase-resolved SFG measurements
- Droplet freezing assays
- Trigonal order in CHL monolayers

Structure of the CHL-water interface as a function of S_A/mol.

It is interesting to investigate the molecular-level details of the CHL-water interface via the molecular dynamics (MD) simulations detailed in the main text. In Fig. S1a we report the number density profile (with respect to the z-axis parallel to the long edge of the simulation box, see Fig. 2a in the main text) of the oxygen atoms within the hydrophilic heads of the cholesterol molecules - for different values of surface area per molecule (S_A/mol): it is evident that for low values of S_A/mol the hydrophilic heads of the CHL molecules span a much wider range of heights (in terms of their positions along the z-axis) compared to what we observe for those values of S_A/mol corresponding to the "most ordered" region described in the main text.

Fig. S1b report instead the number density of oxygen atoms within the water molecules: it can be seen that across the entire range of S_A/mol we have considered in this work,

there exists a region of at least ≈ 4 nm within which the water density is very much bulk-like. This is an essential requirement when investigating the emergence of ice nuclei in the neighbourhoods of the CHL-water interface.

One aspect of simulations contains heterogeneous interfaces and/or molecular species characterised by intrinsic dipole moments is the treatment of the electric field. Ideally we want the electric field within the simulation box to be symmetric with respect to the centre of mass of the CHL bi-layer and it would also be desirable to have a value of zero for the electric field within the vacuum region of the simulation box. As shown in Fig. S1c, our setup does achieve this situation: it is interesting to note that the magnitude of said electric field is highest within the values of S_A/mol corresponding to the above mentioned most ordered region - a sign of the enhanced order of the water molecules at the CHL-water interface. In fact, this particular trend can be appreciated quite clearly in Fig. S1d, where we report the average value of the angle θ_Z between the z-axis and the dipole moment of the water molecules as a function of S_A/mol.

Finally, we report in Fig. S1e the value of the SMAC order parameter[1] for the CHL molecules as a function of S_A/mol. The idea behind this order parameter is that what differentiates a molecular solid from a molecular liquid is an alignment of internal vectors in neighbouring molecules, so that:

$$SMAC = \frac{\left\{1 - \psi\left[\sum_{j\neq i}\sigma(r_{ij})\right]\right\}\sum_{j\neq i}\sigma(r_{ij})\sum_n K_n(\theta_{ij})}{\sum_{j\neq i}\sigma(r_{ij})}. \quad (1)$$

In this expression (r_{ij}) is the distance between molecule (i) and molecule (j) and $(\sigma(r_{ij}))$ is a switching function that acts on this distance. By including this switching function in the second summation in the numerator and in the denominator we are thus ensuring that we calculate an average over the molecules in the first coordination sphere of molecule (i). The term including (ψ) in the numerator is there to ensure that only those molecules that are attached to a reasonably large number of molecules are taken into account. Lastly, the K_n functions are kernel functions that take the torsion angle, (θ_{ij}), between the internal

orientation vectors for molecules (i) and (j) as input. These kernel functions should be set so that they are equal to one when the relative orientation of the molecules are as they are in the solid and equal to zero otherwise. The final SMAC quantity thus measures whether (on average) the molecules in the first coordination sphere around molecule (i) are oriented as they would be in the solid. Here we have considered the same C_3-C_{25} molecular axis, a single kernel function centred around zero, and six CHL molecules within the first coordination shell. As can be seen in Fig. S1d and Fig. S1e, both θ_Z and SMAC suggest that the CHL islands at the CHL-water interface are already quite ordered for large values of S_A/mol, but they do become more dense and more ordered, more crystalline in fact, when approaching $S_A/mol \sim 35$, right before the collapse takes place - beyond which the order is largely lost.

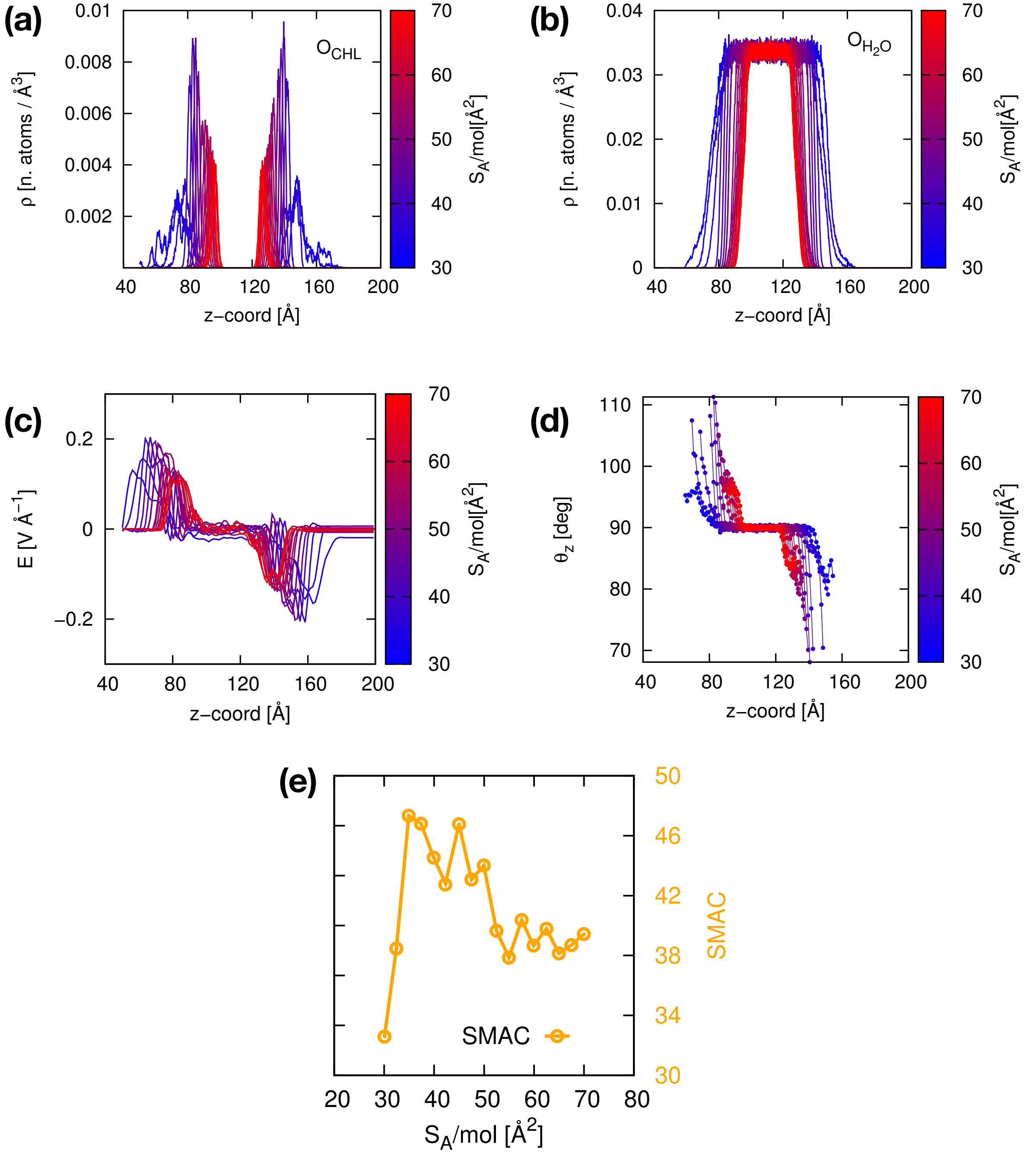

Figure S1: *Structure of the CHL-water interface.* (a) Number density profile (with respect to the z-axis parallel to the long edge of the simulation box, see Fig. 2a in the main text) of the oxygen atoms within the hydrophilic heads of the cholesterol molecules - for different values of surface area per molecule (S_A/mol).(b) Same as (a) but for the oxygen atoms within the water molecules. (c) Electric field along the z-axis within the simulation box as a function of S_A/mol. (d) Average value of the angle θ_Z between the z-axis and the dipole moment of the water molecules as a function of S_A/mol. (e) The SMAC order parameter (see text) for CHL molecules as a function of S_A/mol.

Computational geometry: cholesterol mono- *vs* bi-layers

The computational geometry we have used to obtain the results discussed in the main text has the advantage that, as discussed in the previous section, the system is symmetric with respect to e.g. the electric field within the simulation box. However, the experimental setup involves just one CHL monolayer in contact with water - as opposed to the bilayer we have considered so far. It is thus quite necessary to verify that our results hold for a different computational geometry where we consider a single CHL monolayer in contact with a slab of water. The results are summarised in Fig. S2.

We start by looking at the surface pressure as a function of S_A/mol: as discussed in the main text, it is essential we capture the onset of the monolayer structure collapse observed experimentally. Indeed, we find that the mono- and bi-layer geometries give very similar results in that respect - and both are consistent with the experimental data, as shown in Fig. S2a. Note that the lower values of surface pressure observed upon the collapse for mono- and bi-layers are due to the fact that it takes more mechanical stress to compress a bi-layer compared to a single layer.

The degree of structural order within the CHL mono- and bi-layer is also a concern, given that it might be different according to the different computational geometries. However, as reported in Fig. S2b, this is not the case: both the θ_Z angle and the SMAC order parameter introduced above are very similar for the two setups. In fact, the same can be said for the degree of order of water molecules (panels c and d of Fig. S2) and the electric field within the simulation box (panels e and f of Fig. S2): while there are obvious asymmetries in these profiles in the case of the mono-layer, these quantities at the CHL-water interface are in excellent agreement with the results we have obtained for the bilayer. Note that in Fig. S2d and Fig. S2f the CHL monolayer is positioned at the right side of the distributions (i.e. $\sim$140 Å), whilst the water-vacuum interface is positioned on the left side.

Thus, we are confident that our results are robust with respect to the choice of the specific computational setup.

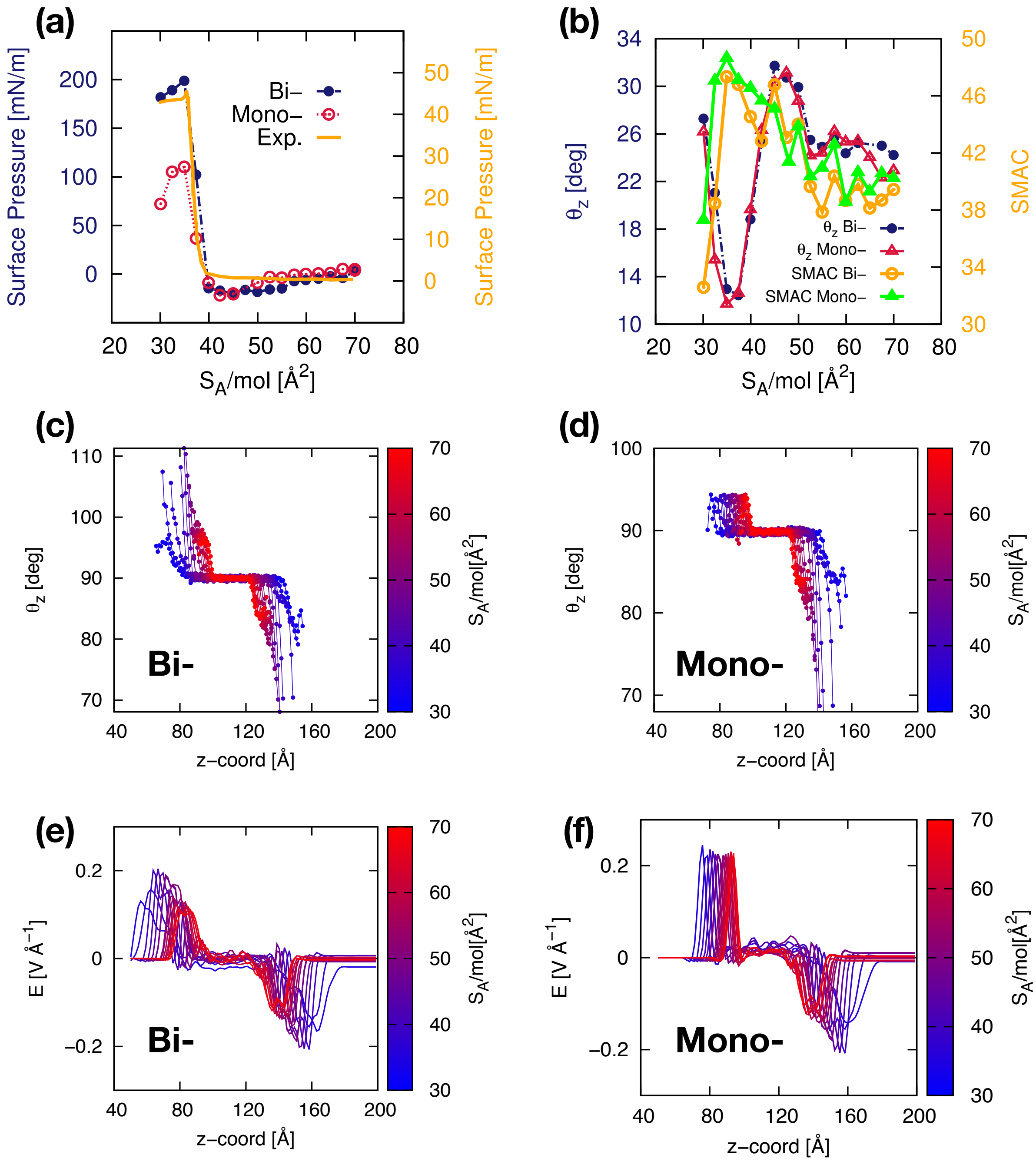

Figure S2: *Cholesterol mono- vs bi-layers.* (a) Surface pressure for CHL monolayers as a function of S_A/mol. Experimental results (Exp.) are compared with our molecular dynamics simulations of mono- (Mono-) and Bi- (Bi-) layers of cholesterol. (b) Average angle θ_Z between the z-axis and the dipole moment of the water molecules (see text) and the SMAC order parameter (see text) for CHL molecules as a function of S_A/mol. (c) and (d) Average value of the angle θ_Z between the z-axis and the dipole moment of the water molecules (see text) as a function of S_A/mol for Bi- (Bi-) and mono- (Mono-) layers of cholesterol. (e) (f) Electric field along the z-axis within the simulation box as a function of S_A/mol for Bi- (Bi-) and mono- (Mono-) layers of cholesterol. Note that in panels (d) and (f) the CHL monolayer is positioned at the right side of the distributions (i.e. ∼140 Å), whilst the water-vacuum interface is positioned on the left side.

Experimental measurements of the surface pressure as a function of S_A/mol.

We report in Fig. S3 a set of independent experimental measurements of the surface pressure as a function of S_A/mol for CHL monolayers. We observe a substantial variability of the results, which were collected using the same instrumentation but at different points in time and using different samples. In particular, we note the emergence of negative surface pressure values, which however we did observe for water in absence of any CHL as well. As such, this effect bears no physical insight and it is probably due to evaporation (especially accelerated when purging) instead. However, we also note that, crucially, the onset of the CHL monolayers collapse is consistent throughout the different measurements - and indeed this value of S_A/mol is in excellent agreement with the molecular dynamics simulations discussed in the main text.

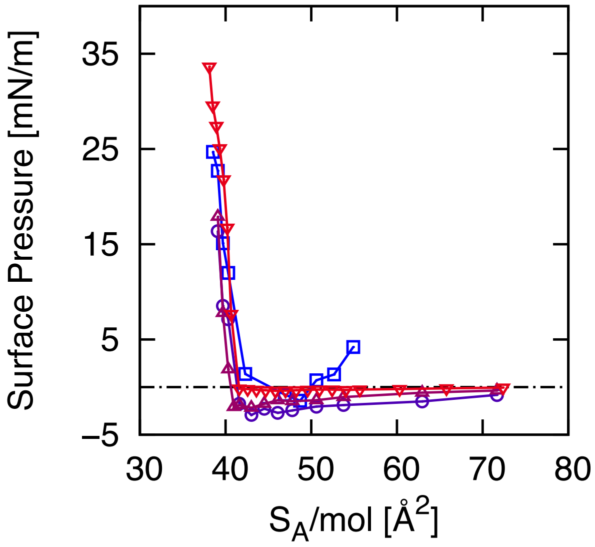

Figure S3: Experimental measurements of the surface pressure as a function of S_A/mol for CHL monolayers.

Brewster Angle Microscopy Measurements

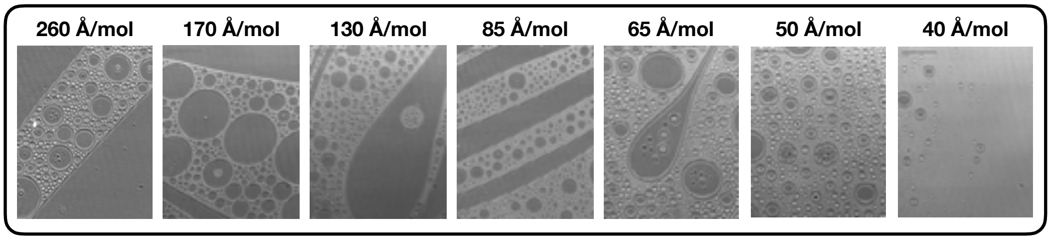

Figure S4: Brewster Angle Microscopy images at different surface coverages of CHL monolayers on water. Darker and lighter regions correspond to water and cholesterol patches, respectively.

Brewster Angle Microscopy (from Nanofilm Surface Analysis) enables visualisation of monolayers at the air-water interface. Bare water surface shows as a dark surface due to no reflection of light, and monolayers show in a brighter form depending on the changed refractive index with the added sample. The images reported in Fig. S4 confirm the increasing coverage of the molecules on the surface of water, and an almost full coverage around 40 $Å^2$/mol.

SFG spectra as a function of CHL coverage

Fig. S5 shows SFG spectra for various CHL coverages. The intensity of the SFG signal is anti-correlated with the decrease of the S_A/mol until it reaches the sweet spot (discussed at length in the main text) around 40 Å^2/mol, below which the SFG intensity reduces.

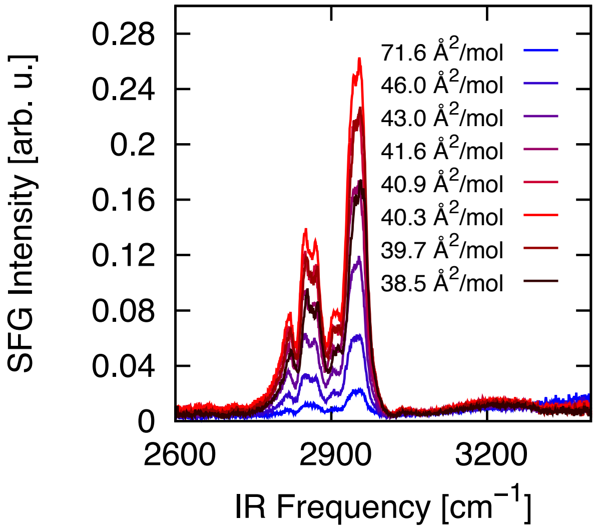

Figure S5: SFG spectra for various CHL coverages.

Phase-resolved SFG measurements

Fig. S6 shows the imaginary $\chi^{(2)}$ spectra, which are indicative of the orientation of the measured sample in reference to the orientation of a known sample. The black curve represents water. The negative amplitude at 3300 cm^{-1} shows that the water molecules point with the hydrogen atoms down, that is towards bulk water. For reference, we also measured a spectrum of DPTAP molecules on water. DPTAP is a long-chain alkyl lipid, and hence gives a clear sign of the alkyl chain direction. DPTAP - shown in blue - gives a very strong negative peak in the water region, as it is positively charged and hence strongly aligns the water molecules. The negative peaks in the -CH region, signifying the alkyl chains pointing up, originate from the -CH_2 and -CH_3 symmetric stretches.

The phase-resolved spectrum (see e.g. Ref 2 for experimental details) of CHL on water is shown in red. The intensity in the -OH region decreases, as compared to the water spectrum. The negative signal indicating an on average downward pointing dipole is in good agreement with Fig. 4c, showing that the average angle is above 90°. In fact, the negative amplitude in the -CH region is indicative of the alkyl chain pointing up (away from the water), as was expected, and affirmatively seen from the simulation data. The positive signal around 2980 cm^{-1} could originate from the -CH_3 asymmetric stretch vibration.

Droplet freezing assays

For analysis, data from multiple experimental runs was collated into single fraction frozen plots. Between 215 and 609 droplets were frozen for each CHL concentration. As most of the results for droplets coated with CHL froze at similar temperatures to the chloroform-treated negative control droplets, differential freezing nucleus concentrations, $k(T)$, were calculated according to the procedure described in Ref. 3 to allow subtraction of the chloroform negative control data from the CHL data. Briefly, it was determined that some of the CHL data collected was not distinguishable from the chloroform negative control. In particular,

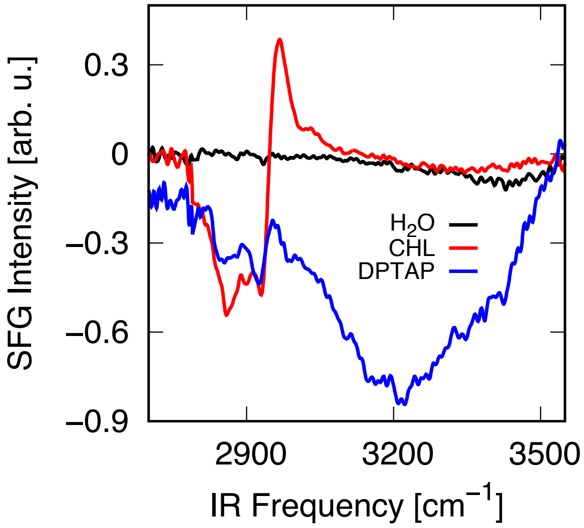

Figure S6: *Phase-resolved SFG measurements.* Imaginary $\chi^{(2)}$ spectra of pure water (black), CHL monolayer on water (red) and DPTAP (blue). As it concerns the CHL spectrum, alkyl chains point away from the water; the intensity in the OH region decreases compared to pure water.

while data for 0.4 and 4 Å/mol are fairly distinct from the background, once the S_A/mol reaches ∼10 Å/mol the CHL monolayer results are practically indistinguishable from the background. For those datasets differing from the background, the cumulative number of sites per cholesterol molecule, referred to as n_s, was calculated according to:

$$n_s = -\frac{\ln[1 - f(T)]}{N}, \tag{2}$$

where $f(T)$ is the fraction of total droplets frozen and N is the number of cholesterol molecules added to the droplet. We chose to normalise to the number of cholesterol molecules per droplet, rather than surface area of nucleant exposed to water, as is more usual, because the nature of the cholesterol/water interface in these experiments is unclear and calculation of a reasonable surface area of cholesterol was not possible. The n_s values should not be over-interpreted physically but allow comparison of the relative effectiveness of the different coverages.

It is important to note that n_s (Fig.1 in the main text) provides a measure of the ice-nucleating activity *per molecule*, whilst the differential spectra (k) reported in Fig. S7 are

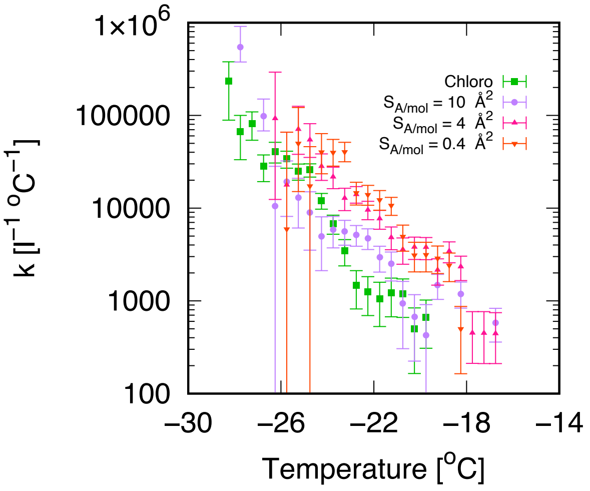

Figure S7: *Droplet freezing assays.* Differential nucleus spectrum (k) for the chloroform "background" along with background-subtracted data for cholesterol monolayers.

not normalised. For instance, the monolayers corresponding to $S_A/mol = 0.4$ Å/mol appear to be the most active according to k. However, once the differential spectra are normalised by the number of molecules, the same coverage turns out to be the least active (see Fig. 1 in the main text). k is not meant to be used to compare the ice-nucleating ability of different systems - but to assess whether the data can be safely distinguished from the background.

Trigonal order in CHL monolayers

It has been reported [4,5] that CHL monolayers on water are characterised by "crystalline" domains characterised by trigonal symmetry. Our simulations are consistent with these observations. At low coverages ($S_A/mol = 62$ Å/mol in Fig. S8a), CHL molecules aggregate in domains that show a certain degree of symmetry, albeit on the timescales (ns) of our simulations we did not observe the emrgence of persistently ordered, crystalline domains. At the surface coverage corresponding to the "sweet spot" in terms of ice-nucleating efficiency ($S_A/mol = 40$ Å/mol in Fig. S8b), the monolayers are as ordered as they can be, and indeed the majority of the CHL phase displays a trigonal symmetry. Finally, when compressed beyond the lift off threshold, Ref. 4 report the emergence of CHL tri-layers, with a rough

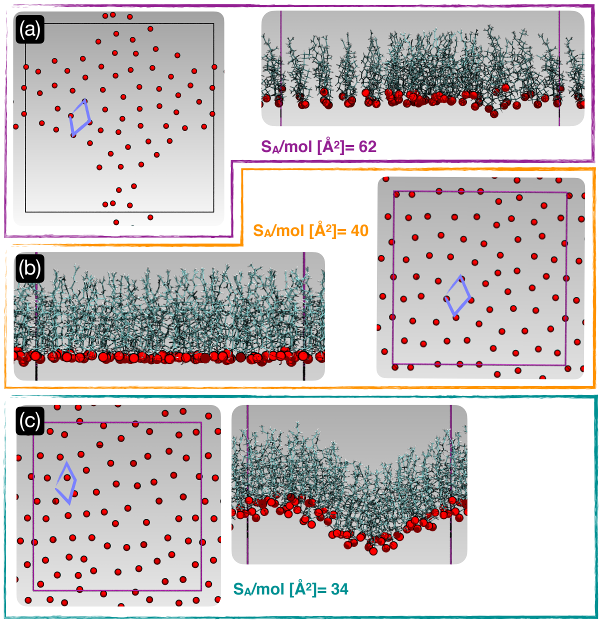

Figure S8: *Trigonal order in CHL monolayers.* Representative snapshots (top and side views) of the simulated CHL monolayers at different values of S_A/mol. Only oxygen atoms (red) belonging to the -OH group of CHL molecules are reported in the top views, whilst the whole CHL molecules are visible in the side views. The trigonal unit cell highlighting the symmetry of the monolayers is sketched in purple

upper surface layer, a smooth, highly crystalline bilayer in the middle, and a more disordered monolayer in contact with the water phase. In our simulations, the limited amount of CHL molecules considered led to a situation where we can only observe a rather disordered monolayer in contact with the water. As illustrated in Fig. S8c (S_A/mol = 34 Å/mol), the CHL molecules are arranged in a rugged monolayer that still retains some degree of trigonal order. Overall, we are thus confident that our simulations are capturing the actual morphology of the CHL monolayers across the entire range of surface coverages considered in this work.

References

(1) Giberti, F.; Salvalaglio, M.; Parrinello, M. Metadynamics studies of crystal nucleation. *IUCrJ* **2015**, *2*, 256–266.

(2) Khatib, R.; Backus, E. H. G.; Bonn, M.; Perez-Haro, M.-J.; Gaigeot, M.-P.; Sulpizi, M.

Water orientation and hydrogen-bond structure at the fluorite/water interface. *Scientific Reports* **2016**, *6*, 24287, Number: 1 Publisher: Nature Publishing Group.

(3) Vali, G. Revisiting the differential freezing nucleus spectra derived from drop-freezing experiments: methods of calculation, applications, and confidence limits. *Atmospheric Measurement Techniques* **2019**, *12*, 1219–1231, Publisher: Copernicus GmbH.

(4) Rapaport, H.; Kuzmenko, I.; Lafont, S.; Kjaer, K.; Howes, P. B.; Als-Nielsen, J.; Lahav, M.; Leiserowitz, L. Cholesterol Monohydrate Nucleation in Ultrathin Films on Water. *Biophys. J.* **2001**, *81*, 2729–2736.

(5) Reuter, S.; Hofmann, A. M.; Busse, K.; Frey, H.; Kressler, J. Langmuir and Langmuir Blodgett Films of Multifunctional, Amphiphilic Polyethers with Cholesterol Moieties. *Langmuir* **2011**, *27*, 1978–1989.
